# Evaluation of medical physics resident well‐being and satisfaction across multiple residency programs

**DOI:** 10.1002/acm2.70475

**Published:** 2026-01-27

**Authors:** Jay W. Burmeister, Nrusingh C. Biswal, Joseph Harms, Adam B. Paxton, Ramesh Boggula

**Affiliations:** ^1^ Karmanos Cancer Center, Department of Oncology Wayne State University School of Medicine Detroit Michigan USA; ^2^ Department of Radiation Oncology University of Maryland School of Medicine Baltimore Maryland USA; ^3^ Department of Radiation Oncology WashU Medicine St. Louis Missouri USA; ^4^ Department of Radiation Oncology University of Utah – Huntsman Cancer Institute Salt Lake City Utah USA

**Keywords:** burnout, medical physics residency

## Abstract

A resident satisfaction and well‐being survey was developed and administered within a Multi‐Institutional Journal Club (MIJC) including therapy medical physics residency programs within the Karmanos Cancer Institute, the University of Maryland, the University of Utah, and the University of Alabama‐Birmingham. The survey was designed as a tool for quality improvement and program evaluation within each individual program. Survey items were derived in part from existing well‐established question inventories and included 26 questions, 4 of which were derived from the Maslach Burnout Inventory (MBI) and 12 from the American Psychological Association Work and Well‐being Survey. The survey was administered anonymously via email link annually from 2022 to 2025, and 41 residents responded to the survey during this period. Mean Likert scores for positively keyed survey items (higher score is better) ranged from 4.00/5 to 4.78/5. Mean Likert scores for negatively keyed survey items (lower score is better) ranged from 1.37/5 to 2.71/5. Items were subsequently grouped into five themes: “Burnout,” “Work‐Life Balance,” “Interpersonal Relationships,” “Institutional Values,” and “Job Satisfaction.” Mean scores for these themes were universally positive and ranged from 4.55/5 for “Job Satisfaction” to 3.63/5 for “Work‐Life Balance.” For the “Interpersonal Relationships,” “Institutional Values,” and “Job Satisfaction” themes, 11 of 12 survey items had a median Likert score of 5/5. No respondent indicated a Likert score under ‘3’ for any of the items in the “Job Satisfaction” theme, making it the most consistently positive theme of the survey. Free‐text comments were categorized as “Positive,” “Neutral,” or “Negative.” Of 70 total free‐text comments, 25 (36%) were categorized as “Positive,” 39 (56%) as “Neutral” and 6 (9%) as “Negative.” Approximately 20% of respondents felt a strong sense of burnout or emotional exhaustion. However, nearly 90% felt that their program and program faculty made them feel valued and that they would recommend their residency program to trainees looking for a position. These results compare favorably with previously published data for radiation oncology residents and represent a strong positive sentiment about the characteristics of these residency programs and the residency process itself. While stress and difficulties maintaining work/life balance were clearly acknowledged, quantitative and free‐text comments indicate that the positive aspects of residency training substantially outweigh these negative aspects. The survey has provided a substantial amount of information supporting the success and best practices involved in our programs as well as some constructive negative feedback, which can allow us to further improve our respective programs and potentially serve as a model to help improve medical physics residency training throughout our profession.

## 1 INTRODUCTION

The pathway to board‐certified clinical practice in medical physics includes an accredited residency program. While this is not new for our board‐certified physician colleagues, it is a comparatively new development for our profession. While we, as a profession, have devoted substantial effort to defining the core elements of clinical training,^1^ it also important to evaluate the logistical and operational aspects of these programs such that they may be, to the best of our ability, optimized to the benefit of our profession, our trainees, and ultimately, our patients. Two such operational aspects that many programs are currently considering are (1) the implementation of collaborative opportunities which can potentially improve the effectiveness of residency training; and (2) the evaluation of resident well‐being, which can potentially improve the effectiveness of our residents. In this work, we report on an instrument which involves both of these operational aspects, specifically, the results of a residency satisfaction survey within a multi‐institutional journal club (MIJC).

The concept of a MIJC within medical physics residency training was initiated as a pilot program incorporating nine imaging residency programs in the Midwest during the COVID pandemic. A Working Group within the American Association of Physicists in Medicine was subsequently created in 2021 to foster the development of these journal clubs, followed shortly by recruitment and assignment of the first cohort. The therapy residency programs within the Karmanos Cancer Institute, the University of Maryland, and the University of Utah were assigned to an MIJC in 2021 and these three institutions have, by request, remained together in the MIJC since then. In 2022 and 2023, the University of Alabama‐Birmingham program also joined this group. All four programs have residency durations of 2 years in length and the total number of residents at any time in the MIJC over the time period 2021–2025 ranged from 8 to 13. This group has endeavored to use this journal club not only to enhance the training of our residents, but also to assess the health and success of our respective programs and residents. As suggested by AAPM Report 249B, educators have an obligation to create an environment most conducive to the success of the trainee, including the promotion of workplace wellness.^1^

Over recent decades, “burnout” has been identified as a work‐related phenomenon often associated with healthcare professions which can lead to decreased job performance and engagement. Burnout can be defined as a state of severe physical, emotional, and mental exhaustion from prolonged, unmanaged stress, often from work. The International Classification of Diseases ICD‐11 characterizes burnout by (i) feelings of energy depletion or exhaustion, (ii) increased mental distance (cynicism/negativity) from one's job, and (iii) reduced professional effectiveness, and it is recognized by the World Health Organization as an “occupational phenomenon”. Burnout has previously been studied in oncology professions.^2^, ^3^, ^4^, ^5^, ^6^, ^7^, ^8^, ^9^, ^10^, ^11^, ^12^ Residency training is often a stressful component within the career pathway into medical practice. As such, burnout and satisfaction have been evaluated in radiation oncology residency training^7^, ^13^ as well as medical physics residency training.^14^ The latter study was a qualitative investigation which included 32 interviewees involved as either residents or faculty members in an accredited medical physics residency program. The four primary themes emerging from that study were (1) the demanding nature of medical physics residencies; (2) the negative impacts of residency on medical physics residents (MPRs) during training and beyond; (3) strategies MPRs use to cope with residency stress; and (4) the role of professional societies in addressing residency‐related change.^14^ This study encouraged us to initiate our own resident well‐being and satisfaction survey which would provide quantitative data for program evaluation and improvement. One of the programs within our MIJC (Karmanos Cancer Institute) had an existing survey of resident satisfaction that is administered after graduation from the program. However, our faculty have recognized the potential lack of complete transparency that may result from an absence of anonymity. We therefore expanded the content of the existing survey and administered it throughout our MIJC. One of the advantages of administering this survey within the MIJC was the potential for greater transparency in responses due to the anonymity attainable within a larger cohort of respondents. Of course, the downside of this process is the potential lack of specificity of results. Within the administration and evaluation of the survey, we attempted to balance these two competing aspects. The purpose of this manuscript is to provide a description of the survey instrument and an analysis of the accumulated data from its administration. Our intent is to provide an example instrument that other programs or groups of programs may wish to adopt or incorporate into their existing quality improvement structure for the benefit of their programs and trainees.

## 2 METHODS AND MATERIALS

A preliminary set of questions for the survey was derived from existing well‐established question inventories, followed by the addition of questions developed specifically within our programs. Our current survey consists of 26 questions, 4 of which were derived from the Maslach Burnout Inventory (MBI)^15^ and 12 from the American Psychological Association 2021 Work and Well‐being Survey.^16^ The MBI is a psychological assessment instrument designed to evaluate occupational burnout through assessment of emotional exhaustion, depersonalization, and personal accomplishment.^15^ Only a small subset of the MBI and APA questions were used within our survey to make it useful, but not overly time‐consuming for our residents. Specifically, MBI questions were chosen only from the ‘emotional exhaustion’ group and APA questions from the ‘work life’, ‘well‐being’, and ‘employer’ sections. However, the survey does preserve core elements of “burnout” including emotional exhaustion, depersonalization/cynicism, and reduced personal accomplishment. Specifically, questions 1, 2, 4, 19, 22, 23, 24, and 25 address emotional exhaustion, questions 3, 9, 10, and 12 address depersonalization/cynicism, and questions 14, 15, 20, and 21 address reduced personal accomplishment. The remaining 10 questions in our survey were developed to evaluate specific experiences of our residents, specific elements of our programs, and general aspects of the medical physics residency experience. All questions were then vetted by the MIJC residency program leadership, including minor rewording of some questions to assure collection of the desired information. The survey was then developed within Google Forms and delivered to all residents via email link to the survey. Residents were given the option to identify their work location but not their identity. The rationale for this option was that feedback would be most useful if individual program leadership knew which results applied directly to their programs, and since this question was optional, the residents were able to retain anonymity if they chose. Since this survey was designed solely for quality improvement and program evaluation, our institutions did not require IRB review.

The full list of questions is provided in Table 1 along with the question source. Questions 1–4 were adopted directly from the MBI and use a 5‐point Likert scale (1‐Never; 2‐Rather infrequently; 3‐Some of the time; 4‐Quite Often; 5‐Always). This scale was chosen for simplicity instead of the 7‐point quantitative scale used in the original MBI. The remaining questions also used a 5‐point Likert scale but in terms of agreement rather than frequency (1‐Strongly Disagree; 2‐Disagree; 3‐Neither Agree or Disagree; 4‐Agree; 5‐Strongly Agree). These include 12 questions adopted directly or derived from similar questions in the APA Survey and 10 questions developed specifically for this survey. The survey begins with the MBI questions, followed by the APA and MIJC questions in a logical order based on content and characteristic.

**TABLE 1:** Multi‐Institutional Journal Club Well‐Being / Satisfaction Survey Questions. Items 1–4 were adopted from the MBI.^15^ Items 5, 10–12, and 17–24 were derived from the APA Survey.^16^ Items 6–9, 13–16, 25–26 were developed for this MIJC survey.

Item	Question
1	I feel emotionally drained from my work.
2	I feel tired when I get up in the morning and have to face another day on the job.
3	Working all day with people is really a strain for me.
4	I feel burned out from work.
5	The work I do is meaningful.
6	I consider it an honor to have the opportunity to serve our patients.
7	I would choose this career path again.
8	The satisfaction I get from taking care of our patients outweighs the long hours and stress of my work.
9	If I had known that residency training would be like this, I would *NOT* have chosen to enter a residency.
10	I have a positive relationship with my co‐workers.
11	My program and program faculty treat me fairly.
12	My program and program faculty make me feel valued.
13	I have experienced discrimination or harassment within my program.
14	While in this program, an inability to maintain a proper work/life balance has negatively impacted my self‐efficacy.
15	While in this program, a lack of support from my program has negatively impacted my self‐efficacy.
16	I can communicate effectively with my program director and/or faculty about any concerns or issues related to my program.
17	I would recommend this residency program to trainees looking for a position.
18	Overall, I am in good physical health.
19	Overall, I am in good psychological health.
20	The demands of my job interfere with my ability to fulfill family or home responsibilities.
21	My home and family responsibilities interfere with my ability to perform my job well.
22	During my workday, I typically feel tense or stressed out.
23	During the past few months, the hours required by my job have significantly impacted my stress level at work.
24	During the past few months, the workload required by my job has significantly impacted my stress level at work.
25	The medical physics residency is more demanding than I had anticipated.
26	Burnout among medical physics residents may have negative consequences for medical physics as a field due to the inability to attract and retain a diverse and talented workforce.

A total of 41 resident responses were collected from 4 different programs, including 8 in 2022, 11 in 2023, 13 in 2024, and 9 in 2025. All residents in the MIJC responded to the survey each year. The number of residents increased as additional residents were added to these programs, then decreased in 2025 when one of the programs was assigned to another MIJC.

## 3 RESULTS

**FIGURE 1:**
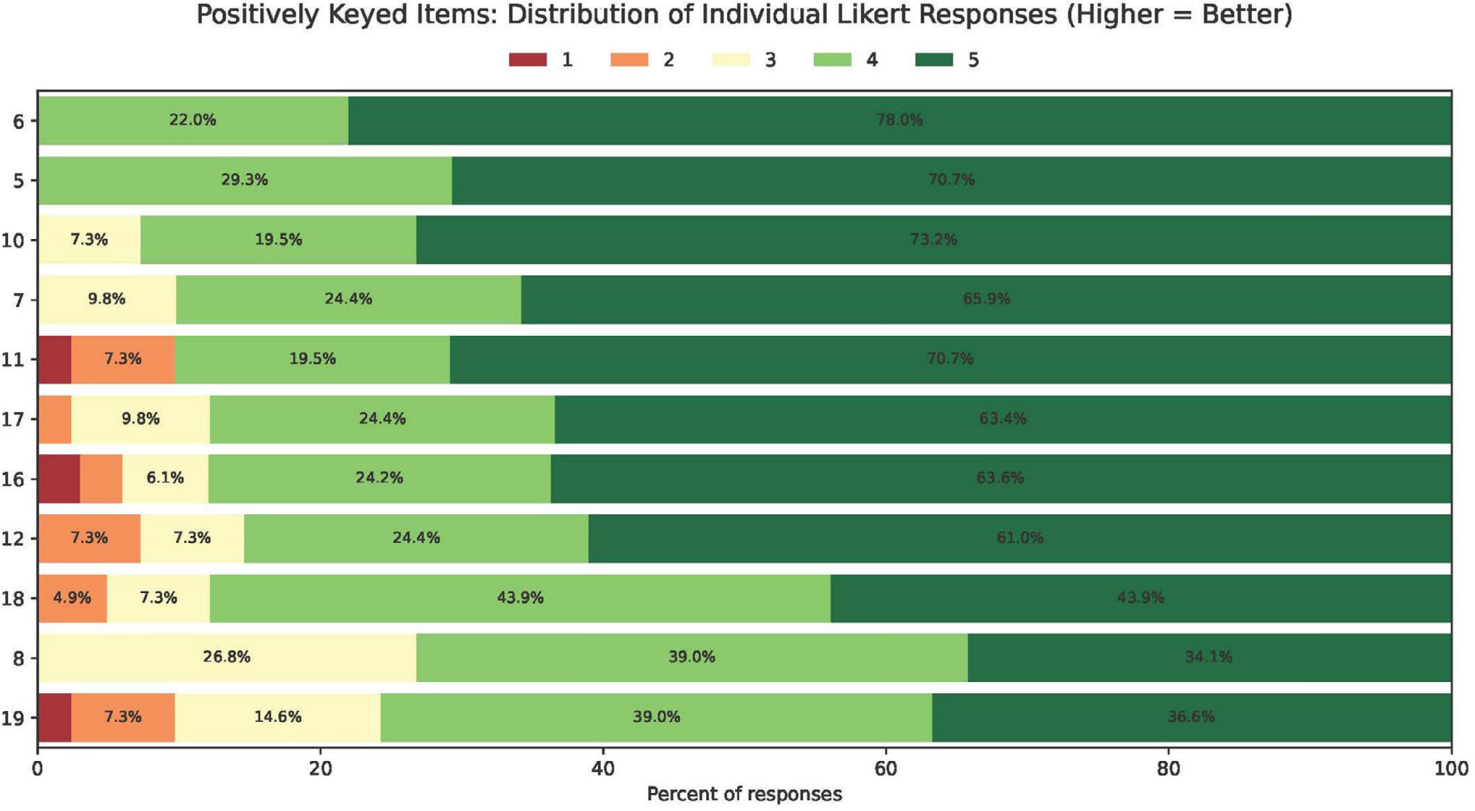
Stacked bar chart of Likert scores for positively keyed survey items (higher score is better), including item number and Likert score percentage, ranked from highest to lowest in order of mean score.

**FIGURE 2:**
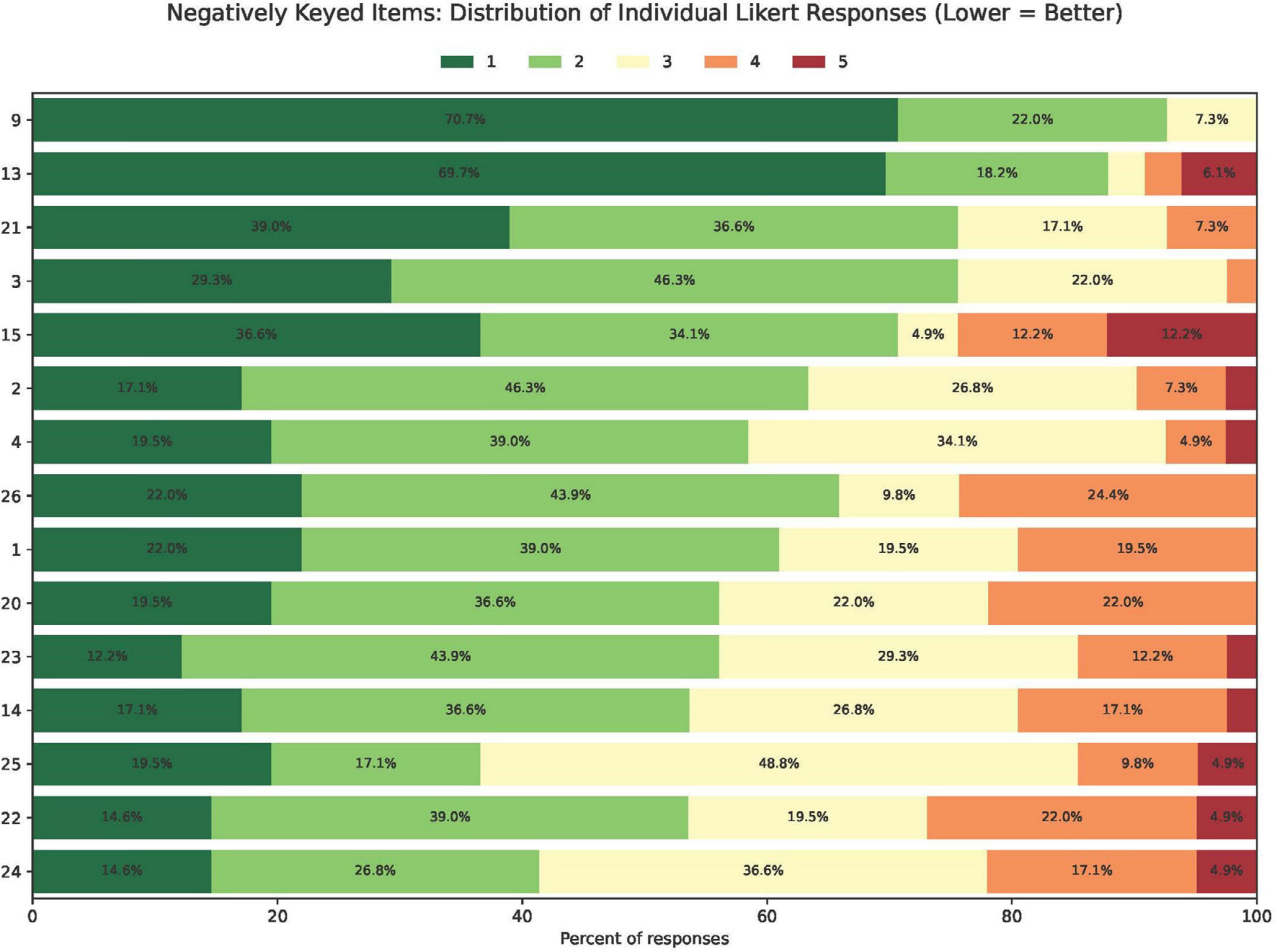
Stacked bar chart of Likert scores for negatively keyed survey items (lower score is better), including item number and Likert score percentage, ranked from lowest to highest in order of mean score.

For quantitative evaluation of the results, all responses were converted into their respective numerical value in the Likert scale. Results are summarized in Tables 2, 3, and 4, which show aggregate values for mean and standard deviation for all responses to each item. Table 2 shows results from positively keyed survey items in which a higher score is better, while Table 3 shows results from negatively keyed survey items in which a lower score is better. These item types were separated to allow visualization of the ranking of each type. Positively keyed items are ranked highest to lowest and negatively keyed items are ranked lowest to highest, therefore ordering both tables from ‘best’ to ’worst’. All Likert data is provided in the form of stacked bar charts for each survey item in Figures 1 and 2. Figure 1 shows Likert scores for positively keyed survey items (higher score is better), including item number and Likert score percentage, ranked from highest to lowest in order of mean score. Figure 2 shows Likert scores for negatively keyed survey items (lower score is better), including item number and Likert score percentage, ranked from lowest to highest in order of mean score. All positively keyed items had a mean Likert value greater than or equal to 4 and all negatively keyed items had a mean Likert value less than 3. Positively keyed items ranged from 4.78 “I consider it an honor to have the opportunity to serve our patients” to 4.00 “Overall, I am in good psychological health.” Negatively keyed items ranged from 2.71 “During the past few months, the workload required by my job has significantly impacted my stress level at work” to 1.37 “If I had known that residency training would be like this, I would *NOT* have chosen to enter a residency.”

**TABLE 2:** Positively keyed survey items (higher score is better), including item number, mean, and standard deviations, ranked from highest to lowest in order of mean score.

Item	Question	Mean	SD
6	I consider it an honor to have the opportunity to serve our patients.	4.78	0.42
5	The work I do is meaningful.	4.71	0.46
10	I have a positive relationship with my co‐workers.	4.66	0.62
7	I would choose this career path again.	4.56	0.67
11	My program and program faculty treat me fairly.	4.49	1.00
17	I would recommend this residency program to trainees looking for a position.	4.49	0.78
16	I can communicate effectively with my program director and/or faculty about any concerns or issues related to my program.	4.42	0.97
12	My program and program faculty make me feel valued.	4.39	0.92
18	Overall, I am in good physical health.	4.27	0.81
8	The satisfaction I get from taking care of our patients outweighs the long hours and stress of my work.	4.07	0.79
19	Overall, I am in good psychological health.	4.00	1.02

**TABLE 3:** Negatively keyed survey items (lower score is better), including item number, mean, and standard deviations, ranked from lowest to highest in order of mean score.

Item	Question	Mean	SD
9	If I had known that residency training would be like this, I would *NOT* have chosen to enter a residency.	1.37	0.62
13	I have experienced discrimination or harassment within my program.	1.58	1.12
21	My home and family responsibilities interfere with my ability to perform my job well.	1.93	0.93
3	Working all day with people is really a strain for me.	1.98	0.79
15	While in this program, a lack of support from my program has negatively impacted my self‐efficacy.	2.29	1.40
2	I feel tired when I get up in the morning and have to face another day on the job.	2.32	0.93
4	I feel burned out from work.	2.32	0.93
26	Burnout among medical physics residents may have negative consequences for medical physics as a field due to the inability to attract and retain a diverse and talented workforce.	2.37	1.09
1	I feel emotionally drained from my work.	2.37	1.04
20	The demands of my job interfere with my ability to fulfill family or home responsibilities.	2.46	1.05
23	During the past few months, the hours required by my job have significantly impacted my stress level at work.	2.49	0.95
14	While in this program, an inability to maintain a proper work/life balance has negatively impacted my self‐efficacy.	2.51	1.05
25	The medical physics residency is more demanding than I had anticipated.	2.63	1.07
22	During my workday, I typically feel tense or stressed out.	2.63	1.13
24	During the past few months, the workload required by my job has significantly impacted my stress level at work.	2.71	1.08

**TABLE 4:** Identified themes along with items contributing to each theme and average mean and standard deviations across each theme.

Theme	Items	Mean	SD
Burnout	1,2,3,4,18,19,22,23,24,26	3.71	1.02
Work/life Balance	14,20,21,25	3.62	1.05
Interpersonal Relationships	10,11,12,13,16	4.48	0.93
Institutional Values	15,17	4.10	1.19
Job Satisfaction	5,6,7,8,9	4.55	0.65

For Table 4, the items were grouped into five themes: “Burnout,” “Work‐Life Balance,” “Interpersonal Relationships,” “Institutional Values,” and “Job Satisfaction.” Providing an average mean for each theme was complicated by the fact that most of these themes had both positive and negative questions. To overcome this limitation, responses for all negative items were transposed such that higher responses are better in all cases. The resulting mean and standard deviation averaged over each theme are provided in Table 4. Average mean values ranged from a high of 4.55 for “Job Satisfaction” to a low of 3.62 for “Work/life Balance.”

All survey items were followed by the following free‐text prompt: “Please add any additional information you would like to provide to clarify your answer to this question:” All free‐text comments were reviewed and categorized as “Positive,” “Neutral,” or “Negative.” Categorization was performed by multiple authors to achieve consensus. Of 70 total free‐text comments, 25 (36%) were categorized as “Positive,” 39 (56%) as “Neutral” and 6 (9%) as “Negative.” “Positive” comments generally spoke to the resident's positive experiences with their program, program leadership, coworkers, profession, and/or amount and nature of their work. “Neutral” comments generally spoke to an anticipated and understood level of difficulty in residency training and/or preparing for a complex profession such as medical physics, and difficulty in meeting their own expectations. “Negative” comments spoke to disparities between perceived clinical expectations and amount of training, long work days, feelings of burnout, and their respective effects on life outside of residency. Representative examples from each category are provided below.

### 3.1 Representative “positive” comments

“I find that the fulfillment which accompanies contributing to patient care outweighs the time spent performing resident duties, even in the cases when some occurrence extends the expected work day significantly. I think the workload that I face is reasonable, especially given how much growth/experience/knowledge needs to be obtained in a two‐year span. Expectations on top of clinical and didactic work (projects, research, etc.) can tip this balance as well, but I find that the faculty are usually reasonable and understanding in providing long time frames to complete these extra tasks.”

“This program strongly supports a healthy work/life balance.”

“I'm very comfortable to message my program director if I have any urgent question, we have weekly meetings. For other faculty, they are always willing to help.”

“I feel supported as a resident and have high work satisfaction.”

“The people here are awesome and always willing to help.”

### 3.2 Representative “neutral” comments

“I often feel frustrated and sad when I cannot answer questions well, keep forgetting things, and realize there is too much for me to learn…But I guess this is mainly because of my own personality (no one blames me or gives me any extra pressure), or maybe learning everything within 2 years is just challenging.”

“A larger workload on a given day somewhat impacts my stress level, but it's a healthy and manageable amount of stress.”

“I am sometimes tired when I work late the night before or went to bed late, but not in a negative context of ‘facing another day on the job’.”

“To be clear, I may be tired and/or drained, but I'm not dreading a day on the job.”

“There are sometimes long tiring days, but nothing I would consider ‘really a strain for me’.”

“Certain departments are overworked, and that can make certain positions miserable. These issues do not impact me, but they do have an impact on the mood of the people around me.”

“I think the main issue, like most programs, is that you get out what you put in.”

“I would say the amount of self‐instruction or seeking out work or help is greater than I expected, but the overall workload has been fine.”

“I've found my role and confidence in the clinic, and it is empowering. The times I fear coming to work are the times when I haven't been adequately trained on something and am expected to take the lead on it. Falling short of expectations makes me feel rather inept despite the fact that I might not have received the necessary training.”

### 3.3 Representative “negative” comments

“I find that, at times, the balance of clinical expectations and reading/didactic expectations is hard to maintain without experiencing “mini‐burnouts,” or needing to rush the reading and thus not having a chance to properly integrate the information contained in the didactic portion of the residency.”

“While hours and days may be longer as a resident, these responsibilities and hours still exist for faculty and staff physics roles, making it a downside of the profession.”

“There are periods where the clinical load happens to skyrocket and there's not much that you can do other than work long days. That means missing out on life and plans, unpredictably. As obvious as this may sound, that lack of work‐life balance in those periods really adds to the burn out effect.”

“There have been periods where not much attention was paid to me and I was expected to know things without any training or communication. Not knowing them when asked and the attitude surrounding my lack of knowledge of that thing certainly shook my confidence and self‐efficacy.”

## 4 DISCUSSION

While acknowledging stress and difficulties maintaining work/life balance, the vast majority of residents indicated a very positive view of their experience in all five themes. All items in the survey had mean values on the positive side of the center of the Likert scale, respectively, and most items (15/26) had mean values greater than or equal to 4 for positively keyed items or less than or equal to 2 for negatively keyed items. Remarkably, the median Likert score was ‘5’ for all 5 items in the “Interpersonal Relationships” theme, both items in the “Institutional Values” theme, and 4 of 5 items in the “Job Satisfaction” theme. These results represent a very strong positive sentiment about the characteristics of these residency programs and the residency process itself. While the “Burnout” and “Work‐Life Balance” themes had lower mean and median Likert scores than the other three themes, only 2 items, “During the past few months, the workload required by my job has significantly impacted my stress level at work” and “The medical physics residency is more demanding than I had anticipated”, had a median Likert score at the midpoint of the Likert scale. In addition, the relatively strong positive responses for items such as “I consider it an honor to have the opportunity to serve our patients”, “The satisfaction I get from taking care of our patients outweighs the long hours and stress of my work”, and “I would choose this career path again” clearly indicate that the positive aspects of residency training substantially outweigh the negative aspects.

While no similar results were identified in the literature for medical physics residents, our results compare favorably to previous studies evaluating these characteristics of residency training in radiation oncology^7^, ^13^ and all oncology specialties.^8^ In our survey, 85% of respondents acknowledged feeling that their “program and program faculty made them feel valued” in comparison to 73% of radiation oncology residents who “felt that faculty and staff cared about their educational success”.^13^ Similarly, 88% of respondents in our survey “would recommend this residency to trainees looking for a position” in comparison to 70% of radiation oncology residents who “would choose the same residency again”.^13^ While we provide these results for comparative purposes, it is difficult to directly compare results since the questions and cohorts differ substantially. For example, a resident may interpret “feeling valued” differently than “faculty caring.” Approximately 20% of respondents to our survey strongly agreed with “I feel burned out at work”, compared to 33% of radiation oncology residents who experienced high burnout level based on the MBI.^7^ While 22% of respondents to our survey strongly agreed with “I feel emotionally drained from my work”, 28% of radiation oncology residents reported high levels of emotional exhaustion^7^ and 42% of oncology residents met burnout criteria defined using an abbreviated version of the MBI.^8^ Finally, 22% of respondents in our survey acknowledged that “the demands of my job interfere with my ability to fulfill family or home responsibilities” compared to 9% of radiation oncology residents who “did not feel that residency allowed for sufficient time for personal life”.^13^ The meaning, intensity, and effects of “interference with responsibilities outside of the job” will differ from one trainee to another. As such, we recommend using such data as a tool to initiate discussion with trainees in an effort to minimize negative effects on life outside of residency while maintaining the resident's ability to gain as much from the training program as possible.

It is not surprising to find substantial stress and potential burnout among medical physics residents, or that quantitative results are similar between our survey of medical physics residents and published results for radiation oncology residents. It is well known that medical physics is a demanding and stressful profession. Indeed, the study by Mazur et al. demonstrated that medical physicists have the highest mental demand and highest workload levels as defined by the National Aeronautics and Space Administration Task‐Load Index.^2^ In contrast, a study by the German Society for Radiooncology demonstrated statistically lower levels of stress for physicists than those of other professional groups working in radiotherapy.^17^ While differences in the nature of work and workplace may differ substantially between these two studies performed in the US and Germany, respectively, the primary difference appears to be the nature of the stressors evaluated. While the former study focused primarily on workload related stressors, the latter study focused substantially on human interactions such as stress by compassion for patients, from interaction with patients, and from problems with colleagues. Physicists scored lowest among all groups in each of these areas.^17^ One should also expect similarities in relative job satisfaction related to the nature of the role of both the radiation oncologist and medical physicist, and this survey certainly supports that hypothesis. In a study of well‐being parameters among academic physicians in 53 different medical specialties, radiation oncologists reported the highest professional fulfillment.^18^ Similarly, the mean Likert score over all “Job Satisfaction” items was 4.55, and no respondent indicated a Likert score under ‘3’ for any of the items in this theme, making it the most consistently positive theme of the survey.

There were a small number of concerning outliers within the survey, including three responses (7%) indicating agreement with the statement “I have experienced discrimination or harassment within my program” and disagreement with the statement “My program and program faculty treat me fairly”. These three responses were very significant outliers since the mean Likert scores for these two items were both over 4.4. In fact, even including these outliers, these two items were among the most positive in the survey. These specific respondents indicated high satisfaction with their work, their co‐workers, and the profession, suggesting that they very much enjoyed their job but had negative feelings about the way they felt treated. They provided no additional details in the free‐text comments and did not identify their program. As a result, each program performed remediation to address the possibility that this sentiment could have come from their program. These remediations were performed immediately after the acquisition of these results and included individual meetings between the program director and each resident, as well as group faculty meetings, in an effort to mitigate potential issues and request additional feedback. No additional feedback was provided to any of the program directors as a result of these meetings. While this made it difficult to remediate these identified issues, it does highlight the utility of this survey in providing a potential mechanism for expression of negative feelings since it allows respondents to retain anonymity. From the subsequent remediation efforts, it appears that these issues would not have been identified without the survey. Thus, any efforts to improve the respective program environments resulting from these remediations are a direct result of the implementation of this survey.

We do recognize the possibility that only one resident, or a small number of residents, may choose to remain anonymous, thus making either their specific responses or their program potentially identifiable. However, only one individual accessed the survey results, preserving anonymity across the remaining faculty and residents throughout the programs. This individual distributed responses to respective program leadership within the other programs. Each program received aggregate summarized data, data from their residents who chose to identify their institution, and any anonymous data. Anonymized aggregate summarized data was also provided to all faculty and residents within the MIJC and discussed within a journal club meeting each year after administration of the survey. Finally, individual programs evaluated their institutional data and the anonymized data and met with faculty and residents to determine a plan of action for potential remediation and follow‐up.

In the previous study by Paradis et al., four main themes were identified, including (1) “the demanding nature of medical physics residencies”; (2) “the negative impacts of residency on MPRs during training and beyond”; (3) “strategies MPRs use to cope with residency‐related stress”; and (4) “the role of professional societies in addressing residency‐related change”.^14^ Our results and free‐text comments do substantiate the demanding nature identified in theme (1), however, the number of positive responses associated with this theme was much greater than the number of negative responses. Our results also suggest a general feeling that this demanding nature is necessary for preparation for such a role, particularly given the brevity of the 2‐year training period. Numerous free text comments shared similarity with the sentiment in the sample comment above “*…I think the workload that I face is reasonable, especially given how much growth/experience/knowledge needs to be obtained in a two‐year span…*” This sentiment seemed substantially different in nature than the responses presented in the study by Paradis et al. In relation to theme (2), our study did support some negative impacts of residency training during the program, but these appeared to be strongly outweighed by the satisfaction associated with the opportunity to practice medical physics. In fact, the remarkably high scores in the job satisfaction theme, including explicit statements about choosing to enter residency again, choosing this career path again, and the meaningful nature of the work, seem to be at odds with “negative impacts of residency on MPRs during training and beyond.” Our study did not explicitly evaluate themes (3) and (4). While free‐text comments acknowledged substantial workload and stress, they also suggested these to be an expected consequence of the comparatively short residency training period. It may be advisable to monitor this sentiment longitudinally as a profession since it may be exacerbated in the future as we continue to expand the breadth of training recommendations.

This work utilized the collaborative opportunity provided by an MIJC to perform an evaluation of medical physics resident well‐being on the participating residents and we recommend implementation of such a survey instrument to other interested MIJCs. Such a mechanism might also be more broadly implemented by the American Association of Physicists in Medicine or the Society of Directors of Academic Medical Physics Programs to gather large‐scale data on burnout. A common theme appears to be that some significant level of stress is expected during a medical physics residency, but the ability for a program to compare its residents’ stress levels relative to other programs may be an important and useful tool. Inclusion of a greater number of participating programs may also help elucidate some of the contrasting findings between this work and that of Paradis et al.

Psychological research on well‐being suggests that it is associated with autonomy, competence, and relatedness.^19^ A recent systematic review has suggested that well‐being in medical residency is similarly associated with these factors.^6^ Free‐text responses from our survey in aggregate suggest that the same holds true for medical physics residencies. In other words, self‐motivated development of proficiency and expertise in an area of specialty, performed within an environment in which one's efforts are valued and appreciated, represents a profound recipe for well‐being, self‐efficacy, and job satisfaction.

## 5 CONCLUSIONS

We implemented a resident satisfaction and well‐being survey within our MIJC. The survey included 2‐year therapy residency programs within the Karmanos Cancer Institute, the University of Maryland, the University of Utah, and the University of Alabama‐Birmingham. Survey items were derived in part from existing well‐established question inventories. The survey was administered annually from 2022–2025, and all 41 residents in these programs during that period responded to the survey.

Mean Likert scores for positive survey items (higher score is better) ranged from 4.00/5 to 4.78/5. Mean Likert scores for negative survey items (lower score is better) ranged from 1.37/5 to 2.71/5. Items were grouped into five themes: “Burnout”, “Work‐Life Balance”, “Interpersonal Relationships”, “Institutional Values”, and “Job Satisfaction”. Mean scores for these themes ranged from a high of 4.55/5 for “Job Satisfaction” to a low of 3.63/5 for “Work‐Life Balance.” Within the themes “Interpersonal Relationships”, “Institutional Values”, and “Job Satisfaction”, 11 of 12 survey items had a median Likert score of 5/5. No respondent indicated a Likert score under ‘3’ for any of the items in the “Job Satisfaction” theme, making it the most consistently positive theme of the survey.

Free‐text comments were categorized as “Positive”, “Neutral”, or “Negative.” Of 70 total free‐text comments, 25 (36%) were categorized as “Positive”, 39 (56%) as “Neutral” and 6 (9%) as “Negative”. “Positive” comments described residents’ positive experiences with their program, program leadership, coworkers, profession, and/or amount and nature of work. “Neutral” comments described an anticipated and understood level of difficulty in residency training and/or preparing for a complex profession such as medical physics, and difficulty in meeting their own expectations. “Negative” comments described disparities between perceived clinical expectations and amount of training, long work days, feelings of burnout, and their respective effects on life outside of residency.

Approximately 20% of respondents felt a strong sense of burnout or emotional exhaustion. However, nearly 90% of respondents felt that their program and program faculty made them feel valued and that they would recommend their residency program. These results compare favorably with previously published data for radiation oncology resident trainees. In summary, these results represent a very strong positive sentiment about the characteristics of these residency programs and the residency process itself. While stress and difficulties maintaining work/life balance were clearly acknowledged in the survey, quantitative and free‐text comments indicate that the positive aspects of residency training substantially outweigh the negative aspects.

The survey has provided a substantial amount of information supporting the success and best practices involved in our programs, as well as some constructive negative feedback, which can allow us to further improve our respective programs and potentially serve as a model to help improve medical physics residency training throughout our profession. A common theme appears to be that some significant level of stress is expected during a medical physics residency, but the ability for a program to compare their residents’ stress levels relative to other programs may be an important and useful tool. The MIJC has not only enhanced the training of our residents, it has also allowed us the ability to assess the health and success of our respective programs and residents through this survey in a way that would not have been possible without it.

## AUTHOR CONTRIBUTIONS

All authors meet the following criteria: Substantial contributions to the conception or design of the work; or the acquisition, analysis, or interpretation of data for the work; and drafting the work or revising it critically for important intellectual content; and final approval of the version to be published; and agreement to be accountable for all aspects of the work in ensuring that questions related to the accuracy or integrity of any part of the work are appropriately investigated and resolved.

## CONFLICT OF INTEREST STATEMENT

The authors declare no conflicts of interest
